# Comparative Genomics of Korean Infectious Bronchitis Viruses (IBVs) and an Animal Model to Evaluate Pathogenicity of IBVs to the Reproductive Organs 

**DOI:** 10.3390/v4112670

**Published:** 2012-10-30

**Authors:** Seung-Min Hong, Hyuk-Joon Kwon, Il-Hwan Kim, Mei-Lan Mo, Jae-Hong Kim

**Affiliations:** 1 Laboratory of Avian Diseases, College of Veterinary Medicine, Seoul National University, Seoul 151-742, Korea; Email: maldalija@gmail.com (S.-M.H.); ilhwan98@snu.ac.kr (I.-H.K.); momeilan@163.com (M.-L.M.); 2 Reseach Institute for Veterinary Science, College of Veterinary Medicine, BK21 for Veterinary Science, Seoul National University, Seoul 151-742, Korea; Email: kwonhj01@snu.ac.kr

**Keywords:** infectious bronchitis virus, comparative genomics, recombinant virus, animal model, ovarian follicle formation

## Abstract

The K-I and nephropathogenic K-II genotypes of infectious bronchitis virus (IBV) have been isolated since 1995 and 1990, respectively, in Korea and commercial inactivated oil-emulsion vaccines containing KM91 (K-II type) and Massachusetts 41 strains have been used in the field. To date, genomic analyses of Korean IBV strains and animal models to test the pathogenicity of Korean IBVs to the reproductive organs have been rare. In the present study, comparative genomics of SNU8067 (K-I type) and KM91 IBVs was performed, and an animal model to test the pathogenicity of SNU8067 was established and applied to vaccine efficacy test. The genome sizes of SNU8067 (27,708 nt) and KM91 (27,626 nt) were slightly different and the nucleotide and amino acid identities of the S1 (79%, 77%), 3a (65%, 52%), and 3b (81%, 72%) genes were lower than those of other genes (94%–97%, 92%–98%). A recombination analysis revealed that SNU8067 was a recombinant virus with a KM91-like backbone except S1, 3a, and 3b genes which might be from an unknown virus. An SNU8067 infection inhibited formation of hierarchal ovarian follicles (80%) and oviduct maturation (50%) in the control group, whereas 70% of vaccinated chickens were protected from lesions.

## 1. Introduction

Infectious bronchitis virus (IBV) is classified into group 3 of the *Gammacoronavirus* genus in *Coronaviridae*. The single-stranded positive-sense RNA genome of IBV is approximately 27 kb and consists of genes coding RNA polymerase/transcriptase, spike protein (S), nonstructural proteins 3a and 3b, proteins E and M, nonstructural proteins 5a and 5b, and nucleocapsid protein (N) [1]. The spike protein is a protective glycoprotein on the virus envelope surface and a determinant of IBV serotype. The spike protein is proteolytically cleaved into S1 and S2. S1 is hypervariable and plays a role in receptor binding, whereas the more conserved S2 functions during fusion of the virus envelope and host cell membrane. The large RNA genome size permits many possibilities of mutations and recombinations which are important mechanisms of viral variation and diversity [2].

IBV replicates in various tissues of chicken and directly or indirectly causes various clinical signs and lesions such as tracheal rales, dyspnea, air sacculitis, diarrhea, proventriculitis, nephritis, atrophic and cystic oviducts, abnormal egg and egg-drop [3]. Symptoms of the respiratory and reproductive systems are common in IBV infection, but several different IBV pathotypes causing nephritis, proventriculitis, and pale and swollen deep pectoral muscles have been reported [4,5,6].

In Korea, IBV field isolates were divided into 3 distinct genotypes, K-I (K-Ia and K-Ib), K-II (K-IIa and K-IIb) and K-III by phylogenetic analyses of partial S1 gene sequences [7,8]. The K-I and K-II viruses have been co-circulating in Korea since the 1990s, and K-II viruses are characterized by a nephropathogenicity [7,9]. A K-III virus which clustered with Chinese strains causing proventriculitis was first isolated in 2005 [7]. K-IIb (QX-type) strains which might have been introduced to Korea from China were first isolated in 2003 and showed different antigenicity from K-IIa (KM91-type) [7,10]. Recently, new recombinant viruses of KM91-type and QX-type strains, and variant strains showing very low amino acid identity (<58.7%) compared to those of previous isolates have been reported [8,11].

To prevent economic losses caused by IBV, an H120-strain live attenuated vaccine and inactivated oil-emulsion vaccines containing both KM91 and Massachusetts 41 (M41) strains have been used in the field. The protective efficacy of a vaccine is dependent on its antigenicity and immunogenicity, and heterologous protection efficacy varies. Virus recovery rates from the trachea, kidney, and/or oviduct in vaccine efficacy tests and the mortality of birds challenged with nephrogenic IBV strain or IBV and *E. coli* coinfection have often been used as immunity criteria [12,13,14]. Although the absence of hierarchal ovarian follicles after experimental IBV infection in specific-pathogen-free (SPF) hens and retardation of laying on layer farms have been reported, experimental data describing the gross lesions of ovarian follicles are insufficient [15].

Although various IBV genotypes have been identified in Korea, studies on comparative genomics and their pathogenicity to reproductive organs have never been performed. Furthermore, continuous isolation of K-I type viruses in the field, even since the popularization of inactivated oil-emulsion vaccination, raise questions about the protective efficacy of commercial vaccines against K-I type viruses. Therefore, in the present study, we performed comparative genomics of SNU8067 (identified here as a K-I type virus) and KM91, and an animal model to test the pathogenicity of SNU8067 to the reproductive organs was established by challenge with SNU8067 in 16-week-old (w-o) specific‑pathogen-free (SPF) hens. The animal experiment was applied to a vaccine efficacy test by challenging SPF hens which had been vaccinated at 13-w-o. We evaluated vaccine efficacy based on a reduction in the number of gross lesions in the ovarian follicles and oviduct.

## 2. Results and Discussion

### 2.1. Complete Genome Sequence Analysis

The complete genomes of KM91 and SNU8067 consisted of 27,625 and 27,708 nucleotides, respectively, and the nucleotide identity rate of the whole genome was 94.5%. The whole genomes revealed 89.6%–89.4%, 89.7%–89.5%, and 86.1%–85.1% identities with M41 (DQ834384), Beaudette (M95169), and partridge/GD/S14/2003 genomes, respectively. The genome sequences of KM91 and SNU8067 were deposited in the GenBank database under the accession numbers, JQ977698 and JQ977697, respectively. The locations of the SNU8067 and KM91 genes and the length of encoded proteins were summarized in Table 1. Most of the gene coding regions overlapped or were linked without non-coding nucleotides, but 359 nucleotides in the non-coding region between genes M and 5a were observed in SNU8067 and KM91 as in other IBV strains (Table 1). The amino acid lengths of 5a, 5b and N were conserved but others were variable between SNU8067 and KM91.Except for the 1b, 5a, 5b, and N genes, the amino acid lengths of other proteins were slightly different between SNU8067 and KM91. 

**Table 1:** Gene of the SNU8067 and KM91 strains in the entire genome.

Gene	Location		The length of protein(aa)	
	SNU8067	KM91	SNU8067	KM91
1a	529–12390	529–12387	3953	3952
1b	12465–20423	12357–20420	2652	2688
S	20374–23883	20371–23862	1169	1163
3a	23883–24056	23862–24008	57	48
3b	24056–24250	23995–24183	64	62
E	24234–24560	24167–24496	108	109
M	24532–25209	24465–25145	225	226
5a	25569–25766	25505–25702	65	65
5b	25763–26011	25699–25947	82	82
N	25954–27183	25890–27119	409	409

The nucleotide and amino acid sequence identities between the IBV strains are summarized in Table 2. Compared to other viruses, SNU8067 showed the highest nucleotide (95%–97%) and amino acid (94%–98%) identities with KM91 in the 1a, 1b, S2, E, M, and N genes. The nucleotide and amino acid identities of S1, 3a, and 3b between compared viruses were 76%–82% and 74%–81%, 65%–95% and 52%–93%, and 81%–93% and 65%–94%, respectively, and the amino acid identities were lower than the nucleotide identities in most cases (Table 2). The 5b gene also showed fewer amino acid identities than nucleotide identities. The nucleotide and amino acid identities of SNU8067 3a and 3b with Connecticut 46 strain were 95% and 93% and 93% and 94%, respectively, and were the highest compared to other strains. In contrast to very low amino acid identities (76%–77%) of SNU8067 S1 with KM91, SAIBK, and the Partridge/GD/S14/2003 strains, those of SNU8067 S2 with KM91, SAIBK, and the Partridge/GD/S14/2003 strains were unexpectedly high (96%–97%) compared to H120, M41, and the Beaudette strains (87%).

**Table 2:** Pair-wise comparison of nucleotide and amino acid sequences of the SNU8067 genes with other infectious bronchitis virus (IBV) strains.

Strain	Nucleotide and amino acid sequence identity (%)										
	1a	1b	S1	S2	3a	3b	E	M	5a	5b	N
Beaudette	90/92 ^a^	92/96	79/78	85/87	8584	86/72	84/89	89/90	92/92	96/92	90/93
ArkDPI11	91/92	94/97	82/81	87/89	91/88	93/92	85/91	91/91	97/97	97/93	93/96
H120	90/91	93/97	79/76	85/87	82/81	86/74	83/91	91/91	93/93	95/89	91/94
SAIBK	87/89	91/96	78/76	94/96	82/74	80/71	86/87	87/89	83/83	94/89	87/93
GD S14	83/85	89/95	79/77	95/96	87/90	73/66	85/88	88/89	84/84	91/88	89/93
Conn46	93/94	93/97	78/76	87/90	**95/93**	**93/94**	85/91	91/91	**97/97**	**98/94**	93/96
M41	91/92	92/96	79/76	86/87	84/79	87/74	84/89	89/91	88/88	96/89	91/94
LX4	84/86	90/96	76/74	90/93	80/71	73/65	84/90	90/91	81/81	91/90	89/94
KM91	**96/96** ^b^	**97/98**	80/77	**97/97**	65/52	81/72	**95/94**	**94/96**	95/95	97/92	**95/96**

^a^ Nucleotide/amino acid identities. ^b^ The highest identity Percentiles among compared genes was represented in boldface.

### 2.2. Phylogenetic Analysis of SNU8067

**Figure 1:**
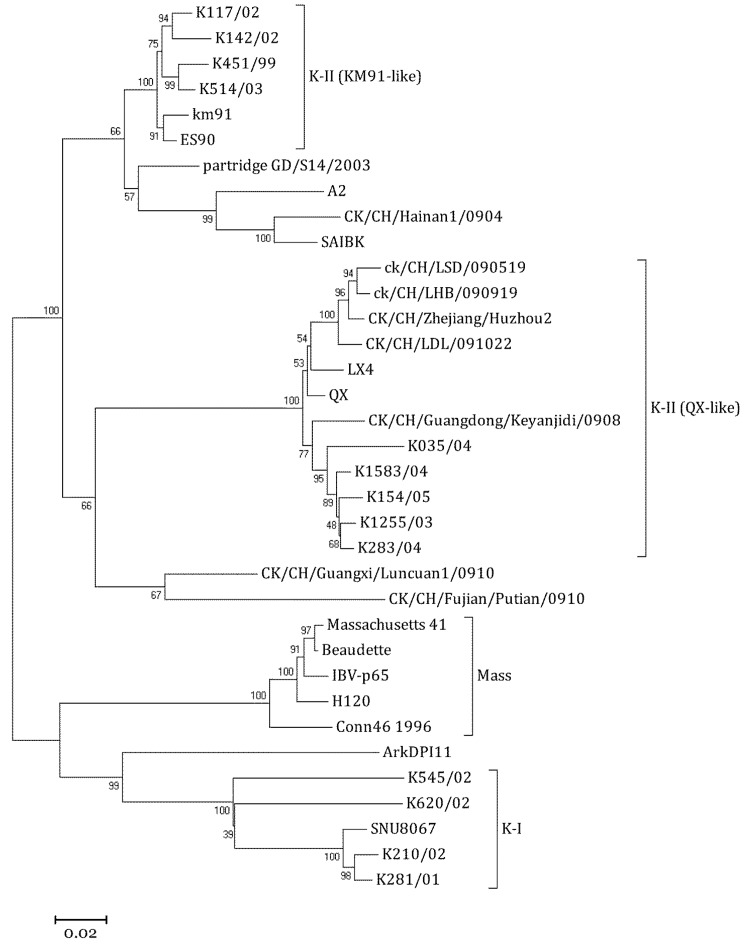
Phylogenetic analysis of the S1 genes of infectious bronchitis viruses. The phylogenetic tree was constructed by the neighbor-joining method (Kimura-2 distance, 500 repeats of bootstrapping).

The phylogenetic analysis of the partial amino acid sequences of the SNU8067 S1 gene clustered with K-I subtype viruses, and KM91 clustered with K-II subtype viruses (Figure 1).

### 2.3. Computational Recombination Analysis

**Figure 2:**
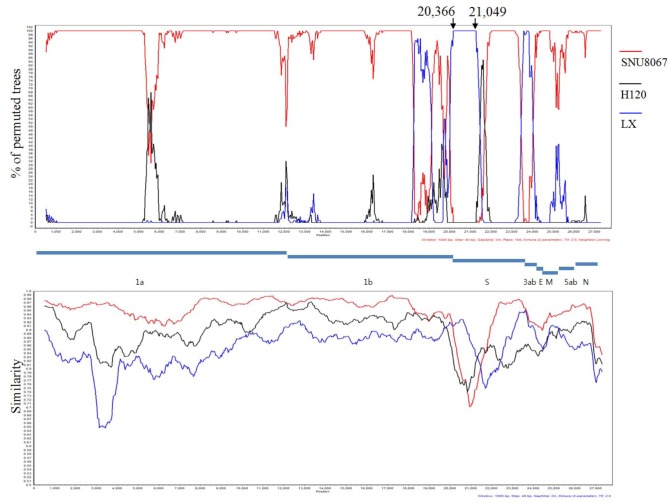
Computational recombination analysis of the KM91, SNU8067, H120, and LX4 strains. The recombination breakpoints were detected by the RDP program (version 4.5) [16] and the similarity and percent of permuted trees were calculated by the SimPlot program (version 3.5.1) [17].

K-I and K-II viruses have co-circulated in Korea for a long time, and H120, an Massachusetts type, attenuated live vaccine strain, has been used as a live vaccine during that time, whereas the QX-type viruses were introduced into the field [7]. Therefore, we tested recombinations between the SNU8067, KM91, H120, and LX4 strains using the RDP and Simplot programs. As a result, the major parent of SNU8067 was KM91; we have compared the S1 sequence to all known S1 sequences in Genbank but did not find a likely source. The KM91 strain contained a small recombination fragment in the S1 gene (20,366–21,049) from the LX4 strain, and a recombination event was supported by the extremely high *P* values for multiple recombination detection methods, such as RDP (2.647 × 10^−78^), GENECONV (1.136 × 10^−63^), BootScan (1.408 × 10^−22^), MaxChi (6.911 × 10^−16^), Chimaera (6.903 × 10^−18^), and SiScan (3.566 × 10^−22^) (Figure 2).

### 2.4. Vaccine Efficacy of a Commercial Inactivated Oil-Emulsion Vaccine against SNU8067

**Figure 3:**
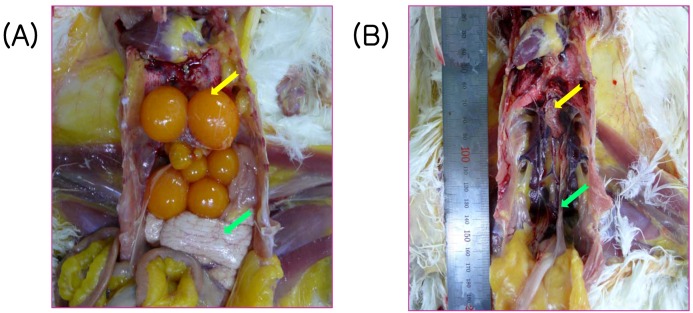
Changes in the ovarian follicles and oviduct of the vaccinated (**A**) and control (**B**) chickens at 5 weeks after challenge infection with IBV SNU8067. Compare the severe aplasia of the ovarian follicles (yellow-colored arrow) and atrophy of oviduct (green-colored arrow) with normal vaccinated chickens.

**Figure 4:**
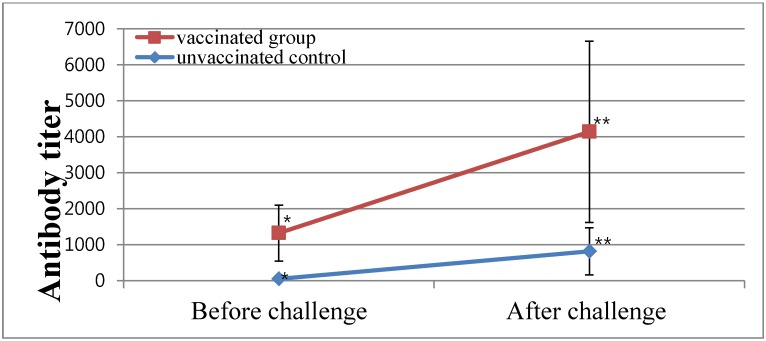
Mean enzyme-linked immunosorbent assay (ELISA) antibody titers before and after challenge in experimental chickens vaccinated with an inactivated IB oil vaccine at 13-week-old and challenged with IBV SNU8067 3 weeks later. The sera were collected at 5 weeks after challenge. The levels of antibody titers between the control and vaccinated groups after vaccination and challenge were significantly different (*P* < 0.05).

The protective efficacy of a commercial oil-emulsion vaccine (BBNE) containing the KM91 and M41 strains against SNU8067 infection of reproductive organs was evaluated by examining the formation of hierarchal oviduct follicles and oviduct maturation. Approximately 70% of the vaccinated chickens were sero-positive for IBV at 3-week-post vaccination, but all chickens in the unvaccinated control group were negative by ELISA. The challenge with SNU8067 (10^6^ EID_50_/100 µL/chicken via ocular route) caused moderate to severe retarded development of hierarchal ovarian follicles in 80% of unvaccinated control chickens and moderate to marked retardation of oviduct maturation in 50% of unvaccinated control chickens. In contrast, only 30% of vaccinated chickens showed moderate to markedly retarded formation of hierarchal ovarian follicles and moderate retardation of oviduct maturation (Table 3; Figure 3). The IBV-specific antibody was detected at 5-weeks post-challenge in the control group and increased in the vaccinated group (Figure 4). The levels of antibody titers between the control and vaccinated groups after vaccination and challenge were significantly different (*P* < 0.05).

**Table 3:** Comparison of gross lesions in the ovarian follicles and oviduct between the vaccinated and control chickens at 5 weeks after challenge infection with IBV SNU8067.

**Challenge group ^a^**	**Number of chickens**	**Lesion score ** **^b^**							
		**Follicle**				**Oviduct**			
		−	+	++	+++	−	+	++	+++
Vaccinated	10	7^c^	1	2	0	8	0	2	0
Non-vaccinated	10	2^c^	2	1	5	5	3	2	0

^a^ A vaccinated group was inoculated intramuscularly at 13-week-old SPF hens with commercial BBNE oil-emulsion vaccine and followed by challenge infection 3 weeks later. ^b^ Lesion score: Aplasia of the ovarian follicles and immature atrophy of the oviduct (−, negative; +, moderate; ++, marked; +++, severe). ^c^ Significantly different (*P* < 0.05).

## 3. Experimental Section

### 3.1. Virus, Eggs, and Chickens

The SNU8067 strain was isolated in 2008 from 11-day-old (d-o) layer chicks (Hy-Line brown) with an unknown vaccine program. The infected flocks showed mortality and respiratory signs and it was isolated from trachea and cecal tonsil samples. KM91 was isolated from broiler chickens which suffered from nephropathogenic IB in 1991 and KM91 has been used with the M41 strain for commercial inactivated oil-emulsion vaccines. KM91 and M41 were kindly provided by the National Veterinary Research and Quarantine Service, Korea. KM91 was inoculated into 10-d-o SPF embryonated chicken eggs (ECE; ValoBioMedia, Adel, IA, USA) via the allantoic cavity route and incubated for 48 h, after which they were chilled at 4 °C overnight. The allantoic fluid containing KM91 was harvested, and the supernatant was stored at −70 °C after centrifugation at 1,500 g for 10 min. Seven passages of SNU8067 were conducted through 10-d-o ECEs until dwarfism and death of embryos were observed. SNU8067 (E7) was harvested and stored at −70 °C until use.

### 3.2. Virus Titration

The SNU8067 titer was determined by inoculating 10-fold serial dilutions (10^−1^–10^−7^) of the virus into five 10-d-o SPF ECE via the allantoic cavity. The inoculated ECE were observed for death and dwarfism for 5 days and the 50% chicken embryo infectious dose (EID_50_/mL) was calculated using the Spearman–Karber method [18].

### 3.3. RNA Extraction and Reverse Transcription Polymerase Chain Reaction (RT-PCR)

Viral genomic RNA was extracted from allantoic fluid using the Viral Gene-Spin kit (iNtRON Biotechnology, Seongnam, Korea) and RT-PCR was carried out using a One-step RT-PCR kit (Qiagen GmbH, Hilden, Germany), according to the manufacturer’s protocols. The cDNA was synthesized at 50 °C for 30 min and then heated at 95 °C for 15 min to activate Taq polymerase and the PCR was performed with 40 repetitions of denaturation at 94 °C for 30 s, annealing at 50 °C for 30 s, extension at 72 °C for 2 min, and a final extension at 72 °C for 5 min. The primer sets for one-step RT-PCR of 19 overlapping fragments of the IBV genomes were listed in Table 4. The accession numbers of the reference strains used for analyses were as follows: Beaudette (M95169 and NC_001451), California 99 (AY514485), M41 (AY851295 and DQ834384), Partridge/GD/S14/2003 (AY646283), Peafowl/GD/KQ6/2003 (AY641576), SAIBK (DQ288927), ArkDPI11 (EU418976), H120 (FJ807652), Conn46 (FJ904716), and LX4 (AY338732).

### 3.4. Sequencing and Genome Sequence Analysis

The PCR amplicons were purified using a PCR purification kit (Macrogen Co. Seoul, Korea) and sequenced with PCR primers using a ABI3711 automatic sequencer (Macrogen). A phylogenetic tree was constructed using MEGA software (version 5.0.5) [19]. The determined overlapping sequences of the 19 gene fragments were assembled into a single complete genome sequence using ChromasPro version 1.5 (Technelysium Pty Ltd., Brisbane, Australia) [20]. The SNU8067 and KM91 genome sequences were aligned with Clustal W in MEGA software (version 5.0.5) [19]. Nucleotide and amino acid identities, variable nucleotide comparisons, and translation of IBV genes were performed with Bioedit software (version 7.0.5.3.) [21]. Phylogenetic analyses, with the complete S1 gene of the IBV strains, were conducted using MEGA software (version 5.0.5) [19], neighbor-joining method with Tamura-Nei distance and 1,000 repeats of bootstrapping) [19]. Putative recombinations between the SNU8067, KM91, LX4, and H120 strains were analyzed with RDP (version 4.5; neighbor-joining, Kimura-distance measure method; *P* < 0.05) [16] and SimPlot (version 3.5.1; Kimura 2-parameter method with transition-transversion ratio of 2 in each window size of 1,000 and step size of 40 with 90% consensus) software [17,22].

**Table 4:** The oligonucleotide primers used for amplification and sequencing of the complete SNU8067 and KM91 genomes.

Primer	Length (bp)	Primer for PCR			
		Position ^a^	Forward-5'	position	Reverse-3'
1	1494	1–26	ACTTAAGATAGATATTAATATATATC	1476–1494	TCAGCTTTTTGRTCAAGCA
2	2474	1247–1265	GGAACTTGTCTTGCAAGYA	3702–3720	CCACAAAAGTCTGCAATTG
3	1595	3623–3642	GCATTGGAYGARTTTAAAGA	5198–5217	TCAACAACTGATTTRATACC
4	1457	5045–5064	GYCAGTTTTGAYRATCTTAC	6484–6501	AGCYCRCCAGCAACTTCA
5	1812	6298–6317	GGATRTAACATGTGAAGTKT	8090–8109	TCATTAAAYAAMACCCATTG
6	1361	8027–8046	AGCTATTGTAGRGGTAGTGT	9368–9387	CCAGTGTGTAATGCATTAGG
7	1458	9244–9263	CCCTGTYACTATGCGYTCTA	10682–10701	GTTGTRCAYTTTACATCACT
8	1586	10547–10567	GATCAATATAGGTATATGTGT	12113–12132	GCTATRTGKGCTCTACAATA
9	1540	11993–12012	CAACCTTTAGGTAAYTGTGT	13513–13532	AARCAAGAMGTTCTAAGATC
10	1549	13366–13385	ATAGCTACTTGTGGCTATCA	14897–14914	GCTCCATAACAGCYACAG
11	1688	14653–14672	GCCAAACARGGTCTTGTWGC	16321–16340	TCACCTACATACACAACATA
12	1391	16183–16202	AATGACACAGGCAAAAAGTA	17554–17573	GCTYTAGAACCRCAAGAACA
13	1463	17464–17486	GTTTCAGATTGYGTAGTKTTTGT	18907–18926	CGCTTRTAACACTGTGTAGA
14	1521	18683–18702	GAAAYATTCGCACACTRCCA	20184–20203	TTGCGTGCAGHGTTTTTCCA
15	1845	20035–20054	ACAGAGACAAGTTGGCAYGA	21860–21879	CCWGAMACTACAAACTGYTG
16	1799	21581–21599	AAGAGYGRTGGCTCTCGTA	23360–23379	GTAACTAYATCTCCTGCAGT
17	1607	23158–23177	TGCACCTAATGGYATAGTGT	24745–24764	GTAYTATCGCTGCGACAAGA
18	1735	24666–24685	TGWTAGTGTTATGGWGCTTT	26381–26400	GGAATGAARTCCCAACGGAA
19	1220	26256–26275	ATGGTATAGTGTGGGTTGCT	27456–27475	TGSTCTAACTCTAWACTAGC

^a^ Basis of M41 (DQ834384).

### 3.5. Vaccine Efficacy Test for the Inactivated Oil-Emulsion Vaccine against SNU8067

Twenty 13-week-old SPF hens, which were purchased from a company (BioPOA Co., Yongin, Korea), were grouped into vaccine and non-vaccine groups, and each bird in the vaccine group was vaccinated intramuscularly with one dose (500 µL) of a commercial BBNE oil-emulsion vaccine (KBNP Co., Yesan, Korea) containing KM91 and M41. Three weeks after vaccination, both groups were challenged with SNU8067 (10^6^ EID_50_/100 µL/chick) via ocular and intranasal routes. The infected chickens were observed for clinical signs and mortality for 5 weeks after the challenge and were euthanized by cervical dislocation. During the experiment hens were caged in A-type commercial cages, and water and feed were provided *ad libitum*. Gross lesions of the reproductive organs were examined based on hierarchal development of ovarian follicles and oviduct maturation was scored on four grades (negative; positive: moderate, +; marked, ++; severe, +++).

### 3.6. Enzyme-Linked Immunosorbent Assay (ELISA)

Specific antibodies against IBV were detected with an ELISA kit (IDEXX Laboratories Inc., Westbrook, ME, USA), and serum titers were calculated according to the manufacturer’s instructions.

### 3.7. Statistical Analysis

The frequencies of lesions on the ovarian follicles and oviduct of the vaccinated and control groups were compared with the chi-square test and the antibody levels between the control and vaccinated groups after vaccination and challenge were compared with the t-test (95% confidence intervals) using SPSS for Windows, version 18.0 (SPSS, Inc., Chicago, IL, USA) [23].

## 4. Conclusions

Since the first isolation of an IBV in 1980 in Korea, IBVs have caused economic losses due to egg‑drop and mortality by nephritis, and the genetic pool of field IBVs has diversified due to the introduction of new viruses from other countries and recombinations between domestic and newly introduced viruses [9,24,25,26,27]. The continuous isolation of K-I type viruses for several years following nationwide inoculation of commercial vaccines composed of KM91 and M41 encouraged studies to understand the genetic backgrounds of evolution and antigenicity of the K-I type viruses and to test vaccine efficacy of commercial vaccines against the K-I type virus in Korea. The results of comparative genomics in this study revealed relatively high nucleotide and amino acid identities between SNU8067 and KM91 strains in most genes except for those encoding S1, 3a, and 3b. The S1 protein is the most important protective antigen in IBVs, and the acquisition of a new SN8067 S1 gene by recombination with an unknown field virus might be the result of virus selection from humoral immunity pressure induced by KM91 type viruses [28]. The S1 amino acid sequence of SNU8067 was different from those of KM91 and M41 by 23% and 24%, respectively, and it may be a different serotype from KM91 and M41 [29]. However, the cross protection of the vaccine containing KM91 and M41 against SNU8067 infection might have occurred because the recombined S1 of SNU8067 was insufficient to evade antibodies induced by KM91 and M41, or that the relatively high identities of the S2 and N proteins conferred cross-protection ability on the vaccine to some extent [30,31,32]. Some IBV serotypes have high amino acid identities with each other and only small portions of S1 proteins form protective epitopes [30,33]. A study on the monoclonal antibody-resistant IBV variants revealed 6 epitopes (D, 24-60; E, 132-149; C/A/B, 291-398; F, 497-543) in S1 and 1 epitope in S2 (G, 548-574) [30].Considering the similar amino acid sequences of SNU8067 epitopes F in S1 and G in S2 to those of KM91 and the decreased isolation frequency of K-I type viruses in the field, SNU8067 might share similar protective epitopes with KM91. The commercial vaccine is successful at diminishing K-I type viruses with the S gene structure of SNU8067. The recombination site between 20,366 and 21,049 of the KM91 genome contained 226 N-terminal amino acids of the S1 protein and was significantly related to the LX4 strain in China. Epitope D and F are present in the region [30]. KM91 was isolated in 1991, is highly nephropathogenic, and causes high mortality in chicks but has been distinguished from previous Korean IBV isolates [34]. Therefore, we could speculate that the KM91-like viruses in Korea might have originated from China where IBVs exchanged their S1 gene fragments to survive under antibody pressure. However, to demonstrate it further study is required.

The 3a, 3b, 5a, and 5b proteins are nonstructural and are unnecessary for virus replication in cell culture and ECEs [35,36]. However, the titers of mutant viruses defective in some of these proteins decline earlier than those of wild type virus in trachea organ cultures. Thus, their function was postulated to be inhibition of innate immunity [35,36,37]. The selective acquisition of new SNU8067 3a and 3b proteins from an unknown virus with retention of the KM91-like virus S2 in the vicinity reflects their biological importance. In addition to the S1, 3a, and 3b genes, 5b showed lower amino acid identities than nucleotide identities, indicating that non-synonymous nucleotide changes causing amino acid changes might occur. Similarly, this protein pattern has been observed in proteins that play roles in host adaptation and evasion of host immune response [37,38]. Therefore, further studies are necessary to reveal the biological functions of 3a, 3b, and 5b. To date, recombination analyses have only focused on the S1 gene but ours and other comparative genomics studies have revealed multiple recombination sites (S2, 3a, and 3b) in IBV genomes [11,39,40,41]. Therefore, further studies on the recombinations of multiple genes, including S1, S2, 3a, 3b, and 5b may shed light on the evolution of IBV and its pathogenicity under host immunity.

IBV replicates in various cell tissues and produces various pathologic lesions and clinical signs. IBV infections in 1-day-old chicks cause permanent lesions resulting in reduced egg production and egg-drop and abnormal eggs with rough, deformed and soft shells, and watery albumen in laying hens [42]. As described in this study, the SNU8067 infection in 16-week-old SPF hens inhibited formation of hierarchal ovarian follicles in 80% of hens and oviduct maturation in 50% of hens in the control group, suggesting that ovarian follicles are more susceptible than the oviduct, and that IBV infection could reduce egg production by affecting ovarian follicle development [15]. Based on the relatively high frequency of lesions and the significant protection rate in the vaccinated group it can be concluded that the ovarian follicle protection model was valuable to test pathogenicity of the IBV strains and the protection efficacy of IB vaccines. The protection rate (70%) of vaccinated hens against ovarian follicle lesions was not high enough but it might be related to the reported innate limitations of inactivated oil-emulsion vaccines in SPF chickens without priming [43]. The correlation of a positive rate as detected by ELISA and the protection rate of ovarian follicles supports application of an ELISA to monitor the protective antibody in the field.

In conclusion, the ovarian follicle protection model was valuable to test the pathogenicity of and vaccine efficacy against IBV infection. The commercial vaccine was protective against the SNU8067 K-I type virus. Furthermore, the multiple recombination sites observed in this study strongly encourage further studies on other gene recombinations as well as S1 among field isolates of various genotypes.
